# Anticancer peptide Q7 suppresses the growth and migration of human endometrial cancer by inhibiting DHCR24 expression and modulating the AKT-mediated pathway

**DOI:** 10.7150/ijms.78349

**Published:** 2022-11-07

**Authors:** Chia-Hung Chen, Tzu-Hsiang Weng, Kai-Yao Huang, Hui-Ju Kao, Kuang-Wen Liao, Shun-Long Weng

**Affiliations:** 1 Department of Medical Research, Hsinchu MacKay Memorial Hospital, Hsinchu City 30071, Taiwan, ROC.; 2 Department of Obstetrics and Gynecology, MacKay Memorial Hospital, Taipei city 104, Taiwan, ROC.; 3 Department of Medicine, MacKay Medical College, New Taipei City 25245, Taiwan, ROC.; 4 Institute of Molecular Medicine and Bioengineering, National Yang Ming Chiao Tung University, Hsinchu City 30068, Taiwan, ROC.; 5 Department of Biological Science and Technology, National Yang Ming Chiao Tung University, Hsinchu City 30068, Taiwan, ROC.; 6 Department of Obstetrics and Gynecology, Hsinchu MacKay Memorial Hospital, Hsinchu City 30071, Taiwan, ROC.; 7 Mackay Junior College of Medicine, Nursing and Management, Taipei City 11260, Taiwan, ROC.

**Keywords:** endometrial cancer, ACP, anticancer peptides, DHCR24, 24-Dehydrocholesterol Reductase, lipid rafts, AKT pathway, LPPC, lipo-PEI-PEG-complex

## Abstract

Endometrial cancer is one of the most common malignancy affecting women in developed countries. Resection uterus or lesion area is usually the first option for a simple and efficient therapy. Therefore, it is necessary to find a new therapeutic drug to reduce surgery areas to preserve fertility. Anticancer peptides (ACP) are bioactive amino acids with lower toxicity and higher specificity than chemical drugs. This study is to address an ACP, herein named Q7, which could downregulate 24-Dehydrocholesterol Reductase (DHCR24) to disrupt lipid rafts formation, and sequentially affect the AKT signal pathway of HEC-1-A cells to suppress their tumorigenicity such as proliferation and migration. Moreover, lipo-PEI-PEG-complex (LPPC) was used to enhance Q7 anticancer activity* in vitro* and efficiently show its effects on HEC-1-A cells. Furthermore, LPPC-Q7 exhibited a synergistic effect in combination with doxorubicin or paclitaxel. To summarize, Q7 was firstly proved to exhibit an anticancer effect on endometrial cancer cells and combined with LPPC efficiently improved the cytotoxicity of Q7.

## Introduction

Endometrial cancer is an increasingly problematic gynecological cancer and one of the most common malignancy affecting women in developed countries 1. In accordance with the International Agency for Research on Cancer, the incidence rate of endometrial cancer is increasing rapidly compared with 2018 and is estimated to increase by more than 50% worldwide by 2040 2. Endometrial cancer treatment usually involves surgery to remove the uterus and ovaries. Other options are included radiation therapy, drug treatments, and hormone therapy to block hormones that cancer cells rely on 3. In recent years, other arising options might be targeted therapy with medicines that attack specifically the cancer cells, and immunotherapy to help patients' immunity against cancer 4. However, advanced stages patients need surgery and possibly loss precious fertility. Before surgery, adjuvant therapy efficiently inhibits tumor growth and decreases the operation area without removal of the uterus; for young women patients, it seems to be an urgent and unmet need 5-7.

Some literature indicated that abnormal metabolism provided nutrients to enhance the malignancy of tumor cells. Increased cholesterol expression has been reported to be positively associated with a high incidence of cancers such as endometrial, colon, and liver 8-10. Cholesterol-rich lipid rafts and caveolae play important roles in cellular signal transduction 11. In several cancers, including endometrial cancer, changes in cholesterol-rich lipid rafts induce the activation of the PI3K/AKT/mTOR pathway in the malignant process, which is central to modulating cell migration, metabolism, proliferation, and survival 9. Because of the imbalance of signals, cancer cells lead to uncontrolled proliferation, migration, and invasion, resulting in tumor expansion across tissue boundaries and metastasis formation 12-14. Therefore, the key regulators in the PI3K/AKT/mTOR pathway are tightly regulated, and good targets in endometrial cancer for tumor therapy 15-17.

Anticancer peptides (ACP), a kind of bioactive peptide, are very promising medicine that exhibits antitumor activity, usually composed of 5 to 50 amino acid residues. ACP has several advantages containing higher specificity and lower toxicity for normal cells compared with chemical drugs 18, 19. Recently, many studies have showed that ACP performs various mechanisms of action. These studies indicated that ACP contained high hydrophobicity and had a positive charge, selectively killing cancer cells by interacting with anionic cell membrane components of cancer cells 19, 20. Some kinds of ACP were disulfide-rich peptides that disrupted cell membranes, such as Cyclotides 21, 22. Then, some ACP induced mitochondrial apoptosis pathway to cause cancer cell death, such as lactoferricin B and tachyplesin 23, 24. Except for activity at the membrane level, ACP could inhibit angiogenesis and activate dendritic cells to eliminate cancer cells such as human neutrophil peptides 1 (HNP-1) 25. In addition, we successfully implemented the two-step model as a web-based tool, namely iDACP, which was now freely available 26.

The stability and half-life of peptides are considered, especially ACP, whose therapeutic effect on cancers is more important 27, 28. To improve the bioavailability of ACP, an appropriate carrier would be a good solution 29. Polycationic liposome containing PEI and polyethylene glycol complex (LPPC) has a lipid bilayer composed of DOPC and DLPC 30. Previous studies had proved that LPPC enhanced antitumor effects by triggering the rapid penetration of drugs into tumor areas to suppress tumor growth and increase drug cytotoxicity in drug-resistant cancer 31. Moreover, LPPC could rapidly deliver enzymatic proteins into cells and maintain their activities, suggesting that it is a promising new tool for cancer therapy 32.

In this study, we investigated the anticancer mechanism that Q7 was ACP identified by iDACP in human endometrial cancer cells such as cell cycle, caspase cascade, and migration regulators. Furthermore, RNA sequencing analysis (RNA-seq) and western blot were used to address the anticancer mechanism of Q7. Additionally, using LPPC enhanced the cytotoxicity of Q7 on HEC-1-A cells. Moreover, LPPC-Q7 had a synergistic effect with the clinical drug doxorubicin and paclitaxel.

## Materials and methods

### Reagents and chemicals

Lipo-PEI-PEG-complex (LPPC) was provided by the lab of Dr. Kuang-Wen Liao, National Yang Ming Chiao Tung University, Hsinchu, Taiwan. Anticancer peptides (ACP) were synthesized and purchased from AngeneBiotech (Taipei, Taiwan). Phenol, chloroform, RIPA lysis buffer and protease inhibitor were purchased from Bio Basic Inc. (Toronto, Canada). MTT, propidium iodide (PI), and dimethyl sulfoxide (DMSO) were purchased from Sigma-Aldrich Chemicals Co. (St. Louis, MO, USA). Pierce™ ECL Western Blotting Substrate was purchased from Thermo (Thermo Fisher Scientific Inc., Waltham, MA, USA). Myco5A medium, M199 medium, EMEM medium, DMEM medium, RPMI-1640 medium, fetal bovine serum (FBS), PBS buffer solution, sodium pyruvate (100 mM), and 0.05% trypsin-EDTA were purchased from Gibco (Grand Island, NY, USA).

### Cell culture

HEC-1-A cells were cultured with Myco5A medium supplemented with 10% heat-inactivated FBS and 1% penicillin/streptomycin (PS; Biological Industries, Beithaemek, Israel). Human umbilical vein endothelial cells (HUVECs) were cultured with M199 medium supplemented with 10% heat-inactivated FBS, 1% PS, 30 µg/ml endothelial cell growth supplement (Sigma-Aldrich), and 25 U/ml heparin (Sigma-Aldrich). HEK-293 cells were cultured with EMEM supplemented with 10% heat-inactivated FBS, and 1% PS. THP-1 cells were cultured with RPMI-1640 medium supplemented with 10% heat-inactivated FBS, 1% PS, 0.05 mM 2-mercaptoethanol (Sigma-Aldrich). SVEC4-10, NIH3T3, and RAW264.7 cells were cultured with DMEM supplemented with 10% heat-inactivated FBS, and 1% PS. All cell lines were purchased from BCRC, Hsinchu, Taiwan.

### Cytotoxicity of anticancer peptide

Cells were seeded into 96-well tissue culture plates at a concentration of 1×10^4^ cells/200 μl/well overnight. Subsequently, the cells were treated with serial dilutions of ACP, scrambled ACP, or non-ACP. After 48 h of incubation, the cell viability of each line was determined by the MTT colorimetric assay (Sigma-Aldrich). Cell viabilities were plotted as a percentage of the untreated control, and the inhibitory concentration at 50% cell survival (IC_50_) of each peptide treatment was determined from the dose-effect curve.

### Western blot

The cells were lysed on ice with 100 μl of lysis buffer and centrifuged at 13,000 g for 20 min at 4 °C. The lysate proteins were quantified using BCA Protein Assay Reagent. Each sample load comprised 30 μg total proteins per lane and was resolved by 12.5% SDS-PAGE. After being transferred onto the PVDF membranes, the membranes were blocked with 5% skimmed dried milk for 1 h and incubated overnight with primary antibodies, including anti-DHCR24, anti-AKT, anti-pAKT, anti-p-GSK3beta, anti-mTOR, and anti-p-mTOR (dilution: 1:1,000; Thermo); anti-S6K1, anti-p-p53, anti-cyclin D1, and anti-cleaved caspase 3 (dilution: 1:500; Thermo); anti-pS6K1 (dilution: 1:2,000; Thermo); anti-p53 and anti-GSK3beta (dilution: 1:200; Thermo); anti-BAX (dilution: 1:2,000; Thermo); anti-p-FoxO3A, anti-HIF-1α, and anti-actin (dilution: 1:1,500; GeneTex Inc., Irvine, CA, USA). The membrane was then washed and incubated with anti-rabbit IgG secondary antibodies followed by horseradish peroxidase. The protein expressions were visualized with an ECL kit (Pierce, Rockford, IL, USA) according to the manufacturer's instructions. Actin acted as the loading control.

### Cell cycle

Cell cycle was detected by EzCell™ Cell Cycle Analysis kit (BioVision, Inc., CA. USA). HEC-1-A cells (5×10^5^ cells/test) were synchronized in a serum-free medium for 24 h. Subsequently, cells were treated with 100 µM ACP or other control peptides at 37 °C for 24 h. Cells in the control group were treated with equal concentrations of DMEM. Following washing twice with PBS, cells were collected by centrifugation at 400g at 4 °C for 5 min, followed by fixation with 70% ethanol at 4 °C for 30 min. After washing with PBS, cells were stained with PI in a staining solution supplemented with RNase A for 30 min at room temperature. The DNA content of total 10,000 cells and the distribution of cells in the G0/G1, S, and G2/M phases of the cell cycle were determined by BD Accuri^TM^ C6 flow cytometry (BD Biosciences, San Jose, CA, USA). Each experiment was repeated three times.

For complexed with LPPC experiments, the cells were treated with 12.5 µM Q7 for 2 h. Removed the treatments, added the new culture medium, and the cell cycle was further analyzed at a 24-h time point.

### Migration assay

HEC-1-A cells were suspended in a serum-free medium and adjusted to 1×10^5^ cells/ml density. Three hundred microliters of cell suspension were added to the upper chamber of migration well for a 24-well plate (Sigma-Aldrich). On the contrary, the lower chamber was filled with 700 µl DMEM containing 10% FBS. After 24 h culture at 37 °C, the cells from the top of the filter were excluded with a cotton swab, and the cells that had migrated through to the underside of the insert membranes were to allow cell fixation by 500 µl of 100% methanol at -20 °C for 10 min. Then, used 50 µg/ml PI to stain cell for 20 min at dark. After washing with PBS buffer three times, cells in three random separate microscope fields were counted (Olympus, Tokyo, Japan). All experiments were performed in triplicate.

### Wound healing assay

HEC-1-A cells (3×10^4^ cells/70 μl/well) were seeded into a Culture-Insert 2 well (Ibidi, Munich, Germany) overnight. Afterward, inserts were removed, peptides (50 µM) were added, the status of the scratch wounds was monitored using an inverted microscope at 0 h (removal of Culture-Insert) and 24 h, and representative images were collected. The results were presented as the percentage of wound closure, calculated as follows: [wound area (initial) - wound area (final)]/wound area (initial)×100. The wound area was measured using software image J.

### RNA-sequence analysis

HEC-1-A cells (2×10^6^ cells/test) were treated with ACP for 1 h or 6 h, and the total RNA was extracted by using the PureLink^TM^ RNA Mini kit (Thermo). Then, the RNA sequencing was by using the NovaSeq 6000 system (Illumina, CA, USA), and the raw count was estimated and normalized as fragments per kilobase per million (FPKM). The significantly up and down-regulated of differential expression genes (DEGs) were selected (Log_2_ Fold Change > ±1, p-value <.05 or p.adjust<.005). The DEGs are annotated with the different biological regulations, which annotation are based on the GO, GSEA, and enrich KEGG in package “clusterProfiler” in R 33-35. The supporting data are available to researchers in the NCBI Sequence Read Archive, under study accession number PRJNA869854.

### Caspase 3 activity

The cells were seeded into 24-well cultured plates at 2×10^5^ cells/well overnight. The cells were treated with 12.5 µM Q7 or LPPC-Q7 for 2 h. After 2 h treatments, removed treatments and added new culture medium for another 6 h incubation. Caspase-3 activity was determined with anti-cleaved caspase 3 (Cell Signaling Tech., MA, USA) and secondary antibody conjugated FITC (Jackson ImmunoResearch, PA, USA). The green fluorescence expressions were analyzed by C6 flow cytometry (BD Biosciences). Each experiment was repeated three times.

### Combination effect analysis

The combination effect analysis was designed such that HEC-1-A cells (1×10^4^ cells per well) were treated with serial dilutions of LPPC-Q7 combined with 0.5 μM doxorubicin or 3.125 μM paclitaxel for 48 h. Also, the cells were treated with serial dilutions of doxorubicin or paclitaxel combined with 6.25 μM LPPC-Q7 for 48 h. The cell viability was detected by MTT assay. The combination index (CI) value = [(drugA+B)IC_50_/(drugA)IC_50_)] + [(drugA+B)IC_50_/(drugB)IC_50_)]. The treatments were defined as having an additive effect (CI = 1), synergism (CI < 1), or antagonism (CI > 1) 36.

### Statistical analysis

Data were from experiments performed in triplicate and shown as the mean ± standard deviation (SD). Student's *t*-test or one-way ANOVA was used to analyze statistical significance. A *P*-value < 0.05 was considered statistically significant.

## Results

### The obtain and characters of anticancer peptides (ACP)

In our previous study, we successfully developed a web tool (iDACP) for determining ACP and non-ACP 26. Firstly, utilized iDACP tool was used to select the ten candidate ACP (Table S1), and the mechanism of these ACP mostly related to disrupt membrane stability. Then, these ACP were applied to the MTT assay to determine the cytotoxic effect on HEC-1-A and HUVEC cells. Table 1 showed ten ACP all had anticancer activity to inhibit HEC-1-A cell proliferation, but nine of ten ACP (NO. 1~NO. 9) ACP also had toxicity in the normal HUVEC cell. Considering the damage to the normal cell, these ACP were filtered out. Finally, one ACP containing fifteen amino acids was obtained, and the sequence of ACP was EQQQQQQPQNRRFRE (UniProtKB-P82331). The peptides originated from one part of the thylakoid in Pisum sativum (Garden pea), herein named Q7. The anticancer mechanism of Q7 was not clearly understood. Sequentially, the anticancer activities and mechanism of Q7 were evaluated and investigated by the following experiments. Then, scrambled Q7 (sQ7), the control peptide of this study, was also fifteen amino acids like Q7, and its sequence was showed as RQPQNQRQEQFQEQR.

### The cytotoxicity of Q7 against HEC-1-A cells *in vitro*

**Figure 1:**
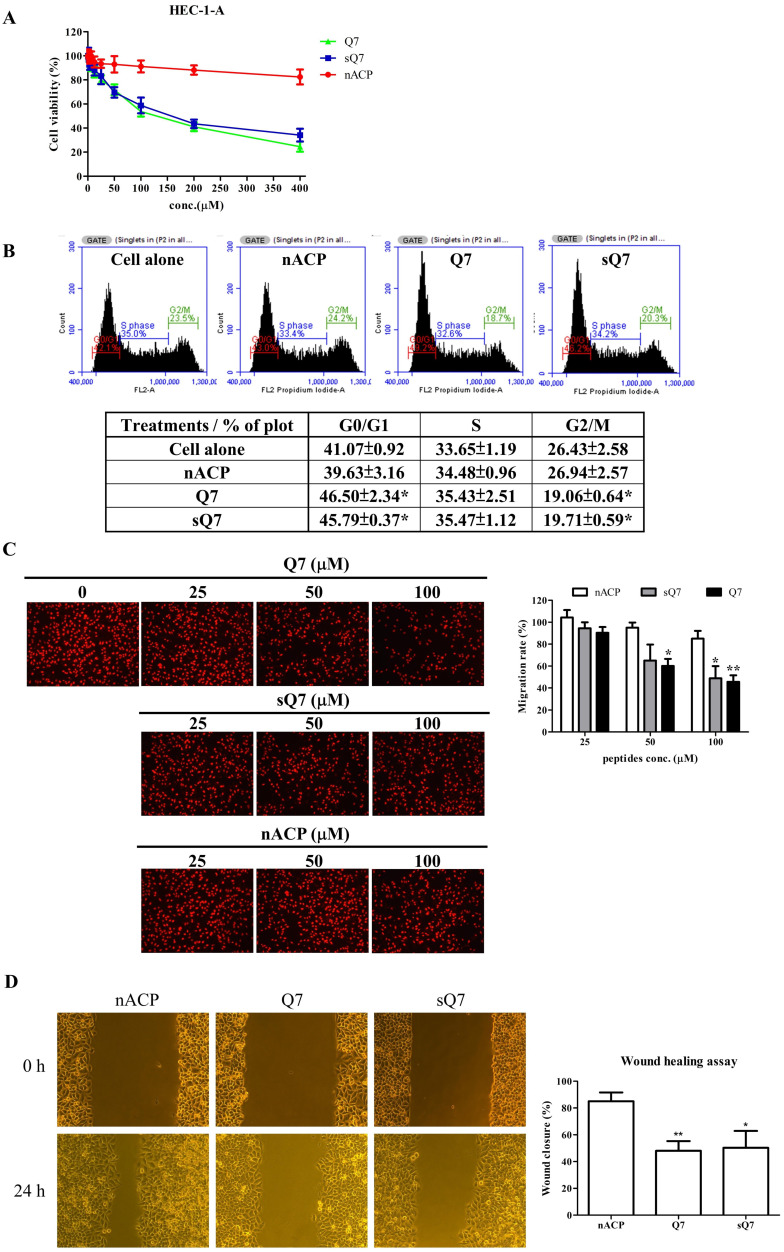
** Q7 inhibited cell proliferation, cell cycle, and migration ability of HEC-1-A cells. (A)** Cell viability of HEC-1-A cells incubated with Q7 (0-400 mM), scrambled Q7 (sQ7), and non-anticancer peptide (nACP). **(B)** After 24 h incubation of Q7 (100 mM), sQ7, and nACP in HEC-1-A cells, the cell cycle was individually analyzed using flow cytometry. The representative results were shown. **(C)** Cells were exposed to peptides in a transwell for 24 h, and the migration rate of HEC-1-A cells was calculated by PI staining. The representative photographs of cells were shown and observed under microscopy (100×). **(D)** Cells were treated with peptides (50 mM), and the wound closure rates of HEC-1-A cells were calculated and shown. *: Indicated a significant difference compared to nACP (p<0.05). **: Indicated a significant difference compared to nACP (p<0.01).

The cytotoxicity of Q7 in the human endometrial cancer cell, HEC-1-A cells, was determined *via* MTT assay treated with increasing concentrations of Q7 (0-400 μM) for 48 h. After 48 h incubation, fifty percent of growth inhibition concentration (IC_50_) values of Q7 and sQ7 individually were 129.82±7.53 μM and 157.66±13.91 μM in HEC-1-A cells (Fig. 1A). The IC_50_ values of Q7 and sQ7 were not significantly different. Furthermore, non-ACP (nACP), human HSP27_36-50_, was not obviously toxic effects on HEC-1-A cells, even 400 μM. In addition, HEC-1-A cells with Q7 or sQ7 treatments were led to clearly different morphological changes. Q7 or sQ7 significantly induced cell membrane shrink and formed apoptotic bodies, whereas the presence in nACP-treated HEC-1-A cells was not as obvious (Fig. S1).

### Effects of Q7 on the cell cycle and migration of HEC-1-A cells

Due to inhibition of cell proliferation and morphological change under the treatments, we furtherly measured the effects of Q7 on the cell cycle and migration. In the cell cycle, HEC-1-A cells were treated with Q7 or other treatments and then analyzed for FL2 intensity by flow cytometry. The result revealed that Q7 could significantly induce cell cycle arrest in G0/G1 phase and along with an obvious percentage decrease in G2/M phase, and sQ7 also did (Fig. 1B). In contrast, treatment with nACP did not cause any significant changes in the cell cycle distribution under the same concentration.

Additionally, the transwell was utilized to analyze the effect of Q7 on the migration ability of HEC-1-A cells. Compared with nACP, the migration rate indicated that Q7 obviously influenced the cell migration activity in a dose-dependent manner (Fig. 1C). Moreover, sQ7 significantly inhibited the cell migration at 100 µM dosage compared with nACP, while a slight difference compared with Q7 but not statistically. Similarly, a culture-insert in wound healing assay was evaluated the activity of Q7. Figure 1D showed that Q7 and sQ7 both significantly inhibited about 50% of cell migration ability compared to the wound closure rate of nACP treatment (85%). Altogether, these data imply that Q7 could suppress HEC-1-A cell growth, migration, and invasion.

### RNA sequencing analysis (RNA-seq) of Q7 treatment for HEC-1-A cells

**Figure 2:**
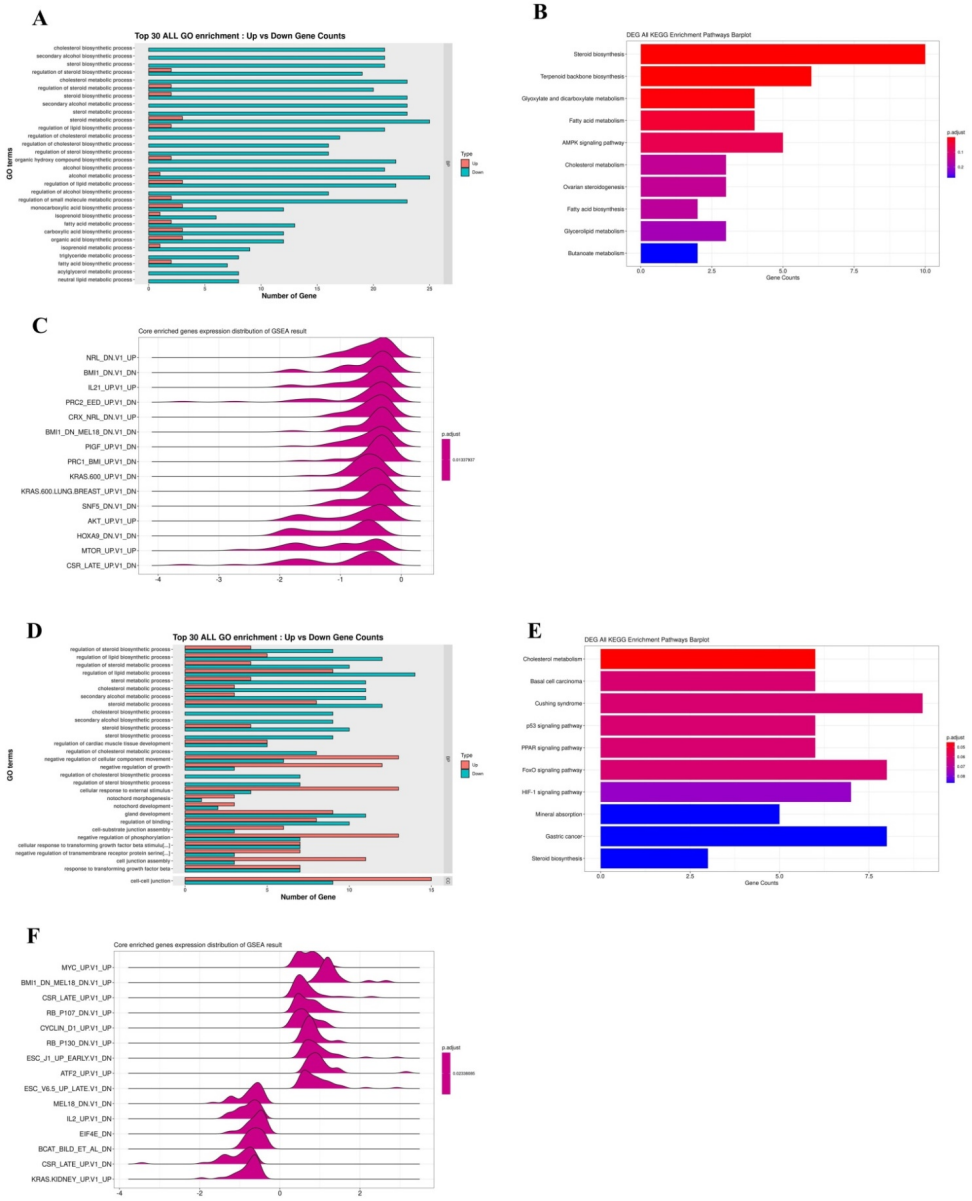
** RNA-seq data analysis for the anticancer mechanism of Q7.** HEC-1-A cells were treated with Q7 for 1 h and 6 h, and the total RNA was further analyzed. In 1-h data, the plots showed the gene function and pathway enrichment significance of DEGs by GO (**A**), KEGG (**B**), and GSEA analysis (**C**); the analysis of 6-h data was also shown (**D-F**).

Furthermore, an RNA-seq tool was used to overview comprehending Q7 effects on HEC-1-A cells. RNA-seq was performed on total RNA of HEC-1-A cells exposed to PBS (control) or treated with Q7 (100 µM) for 1 h and 6 h. After data screening, there were 26 significantly upregulated and 89 significantly downregulated genes at a 1-h time interval of Q7 (Fig. S2A), while there were 302 significantly upregulated and 146 significantly downregulated genes at a 6-h time (Fig. S2C; Log_2_ Fold Change > ±1, p-value <.05 or p.adjust<.005). To determine the possible functions of enriched genes and signal pathways correlated with the anticancer activity of Q7, GO and KEGG enrichment analysis was applied to explore the role of DEGs under Q7 treatment. At the 1-h data, the result demonstrated that the most enriched terms were relevant to the biological process of the downregulation of cholesterol, sterol, and lipid biosynthesis (Fig. 2A and 2B). Then, with the assistance of GSEA analysis, figure 2C indicated tumorgenicity signaling pathways which were enriched involved AKT- and mTOR-associated genes and significantly downregulated. Moreover, these genes were further represented through heatmap in Fig. S2B. Except for transcription factor activity and ion channel, the function of genes in heatmap also included cell proliferation (CDKN2B and FKBP8), biosynthesis of cholesterol (LSS, DHCR24, and DHCR7), cytoskeleton and actin synthesis (TJP3, ALDOC, VILL, PACSIN3, and CLMN), and these genes expressions of Q7 group were inhibited compared with the control group.

Similarly, the 6-h time data sequentially was analyzed by GO, KEGG, and GSEA enrichment analysis, the DEGs related to cell proliferation, migration, and phosphorylation function were enriched, except for cholesterol and lipid biosynthesis (Fig. 2D). DEGs were significantly enriched in p53, FoxO, and HIF-1 signaling pathway (Fig. 2E), and these pathways were modulated by AKT pathway. By KEGG analysis, most DEGs were also involved in AKT/mTOR pathway (Fig. 2F), and these genes were further represented through heatmap in Fig. S2D.

Taken together, these data indicated that Q7 treatment initially downregulated cholesterol-related and lipid biosynthesis; sequentially the cell survival and migration ability of cells were inhibited through the AKT-associated pathway.

### Q7 reduces DHCR24 expression to affect AKT-associated regulators in HEC-1-A cells

Among DEGs of heatmaps under 1-h Q7 treatment (Fig. S2B), DHCR24 was a key gene and some studies suggested that the expression of DHCR24 enzyme could influence membrane lipid-raft organization and stability, which were key factors for cell signaling 37, 38. The lower expression or deficiency of DHCR24 led to cholesterol depletion in lipid rafts of cell membranes. Caveolae, cholesterol-rich lipid-raft microdomains of cell membrane, has been known to mediate the activity of lipid raft-dependent protein kinases, such as AKT kinase. The cellular cholesterol was a pivotal role in the activation of the PI3K/AKT pathway 38-40. As shown in Fig. S3, Q7 and sQ7 both decreased the level of phosphorylation of AKT of HEC-1-A cells, and nACP had no obvious effect like the above results, such as cell cycle and migration assay (Fig. 1). As noted above results, Q7 and sQ7 had similar anticancer effects, therefore, Q7 was applied in the following experiments.

**Figure 3:**
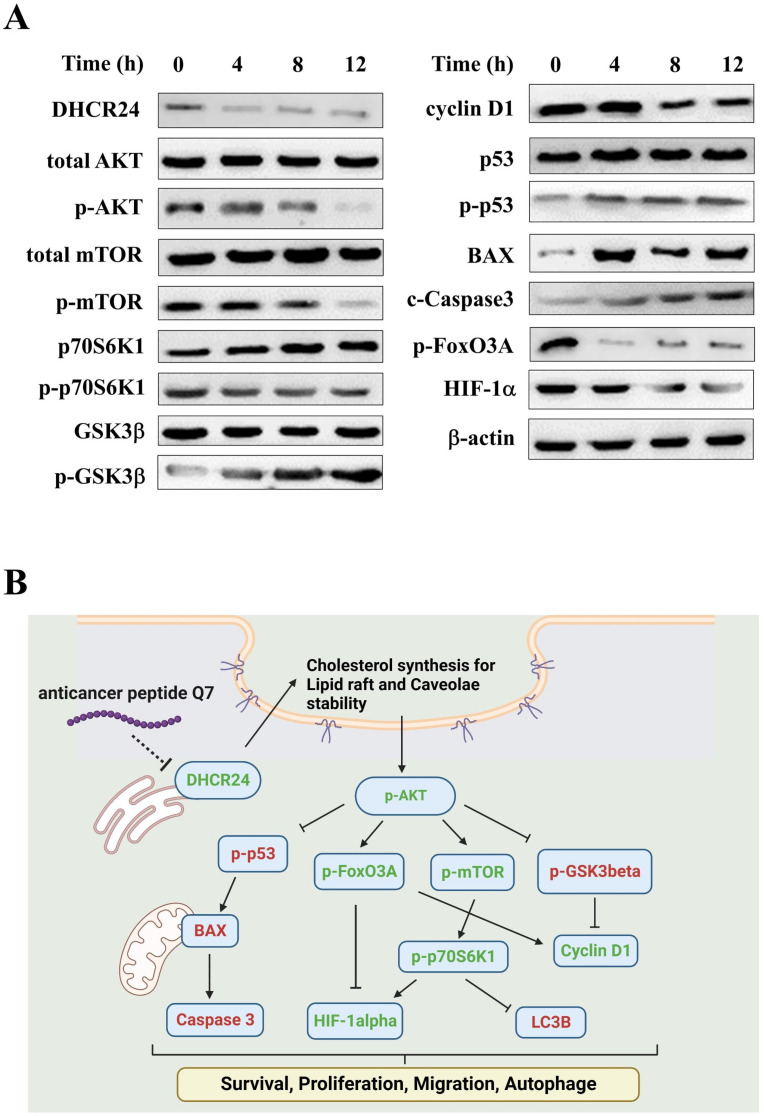
** Anticancer mechanisms of Q7 in HEC-1-A cells. (A)** The western blotting was performed that Q7 influenced migration proteins (mTOR, p70S6K1, and HIF-1a), cell cycle-related proteins (GSk3b, FoxO3A, and cyclin D1), and activated apoptosis proteins (p53, BAX and c-caspase 3) in HEC-1-A cells for different time points (0, 4, 8, and 12 h). **(B)** The picture illustrated that Q7 reduced DHCR24 expression to downregulate the phosphorylation of AKT and the downstream factors that were affected. The speculated mechanisms were created using Biorender (https://biorender.com/), and upregulated proteins expressions were shown in red, and downregulated were shown in green.

Figure S4 showed that the caveolin expression (green fluorescence) of cells in Q7 treatment, the marker of caveolae, was lower than cell alone and nACP treatment. Moreover, DHCR24 protein expression was reduced after Q7 treatment (Fig. 3A). Therefore, Q7 decreased DHCR24 to cause dysfunction of caveolae.

In addition, Figure 3A indicated that the phosphorylation of AKT protein was downregulated under Q7 treatment. To furtherly prove the speculated mechanism, several important regulators were confirmed and evaluated by western blot. Figure 3A revealed that the protein expressions of migration regulators (mTOR, p70S6K1, and HIF-1α) were decreased, while the expressions of apoptosis-related proteins (p53, BAX, and Caspase 3) were increased. Additionally, cell cycle proteins p-GSK3β increased and cyclin D1 decreased after Q7 treatment (Fig. 3A). A central protein in the autophagy marker, LC3B, was increased at 24 h (Fig. S5). Consequently, by the assistance of the RNA-seq data and the western blot results showed that Q7 decreased DHCR24 expression to downregulate phosphorylation of AKT protein to suppress tumorigenicity of HEC-1-A cells and cause cell death. To sum up, figure 3B simply dramatized the anticancer mechanism of Q7 in HEC-1-A cells.

### The cytotoxicity of LPPC-Q7 on HEC-1-A cells

**Figure 4:**
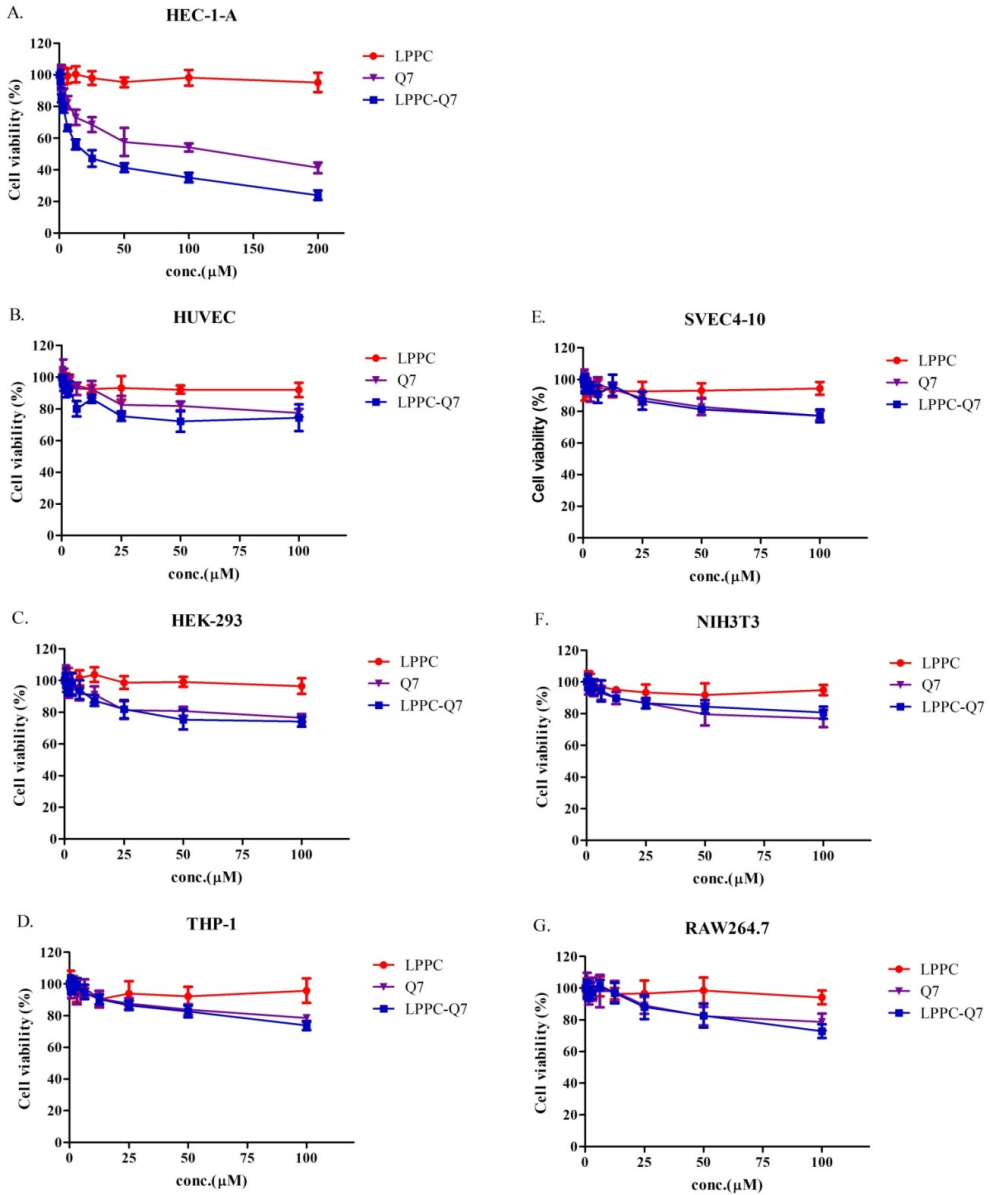
** The cytotoxicity of LPPC-Q7 in HEC-1-A cells and different normal cells. (A)** Cell viability of HEC-1-A cells treatment with Q7 (0-200 mM) or LPPC-Q7 for 48 h was evaluated. **(B-G)** The six normal cell lines were utilized to evaluate the safety of Q7 and LPPC-Q7.

According to previously published studies, LPPC could adsorb, encapsulate, and deliver proteins or drugs into cells to increase their activities 30, 31. Similarly, an MTT assay was used to determine that the cytotoxicity of LPPC-Q7 was on HEC-1-A cells. The result indicated that LPPC-Q7 was able to enhance the activity (IC_50_ values) of Q7 6.5-fold for HEC-1-A cells (Fig. 4A). Moreover, human normal cell lines and other animal normal cell lines were also evaluated the toxicity of Q7 or LPPC-Q7. The results revealed that six normal cell lines under treatments with LPPC-Q7 or Q7 were at least 80% of cell viability (Fig. 4B-4G).

### The rapid delivery of LPPC-Q7 into HEC-1-A cells

**Figure 5:**
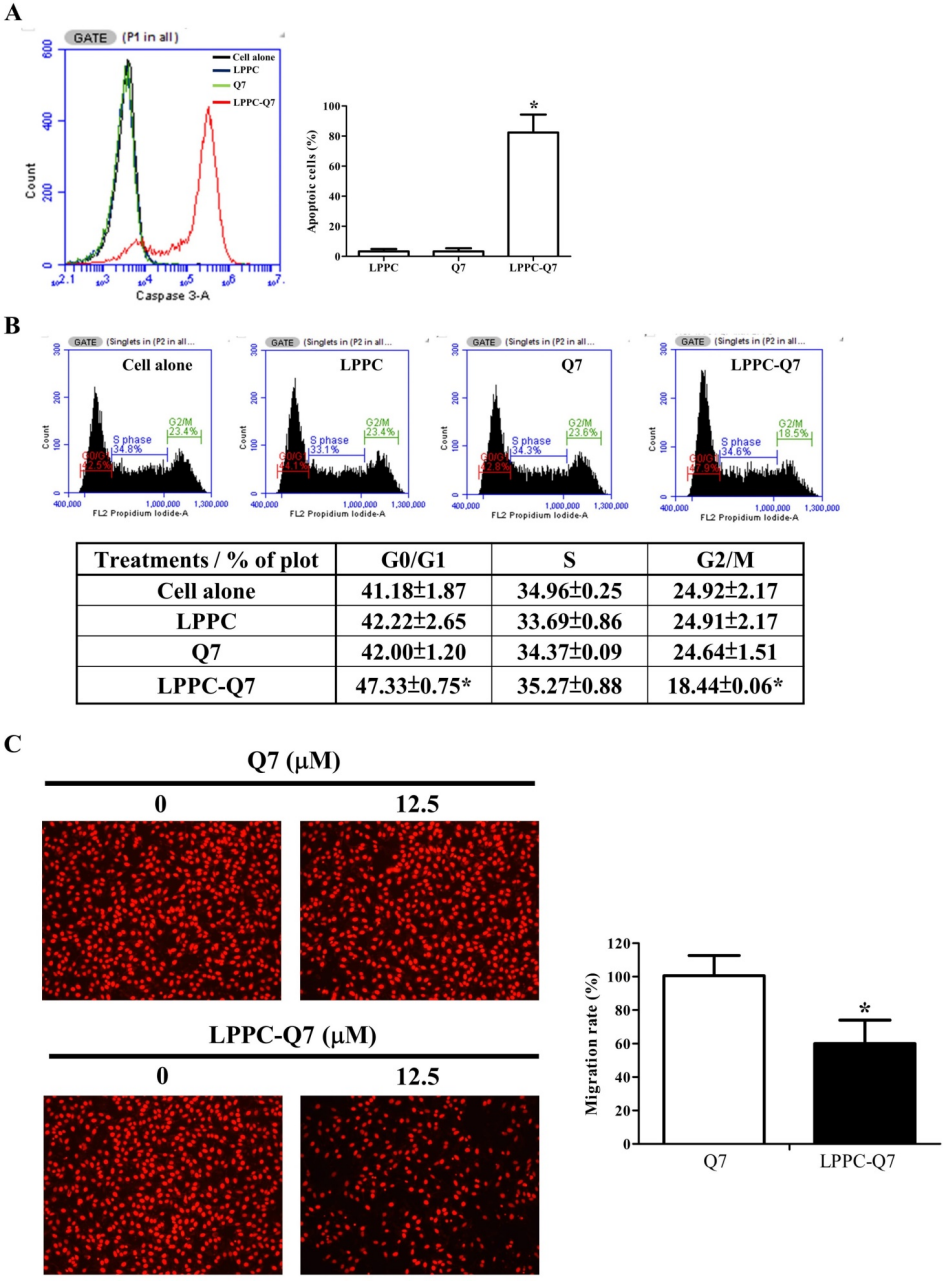
** LPPC assisted Q7 to efficiently provide anticancer effects on the cells.** HEC-1-A cells were pre-treated with Q7 (12.5mM) or LPPC-Q7 for 2 h, and then removed the treatment for following analysis, included caspase 3 expression **(A)**, cell cycle **(B)**, and migration assay **(C)**. The representative results were shown. *: Indicated a significant difference between LPPC-Q7 and Q7 (p<0.05).

Additionally, LPPC had been showed that it delivered proteins into the cells only for 30 min to 2 h and was able to provide the activity with only lower dosage of protein 32. Therefore, after 12.5 μM Q7 of 2-h incubation, and washing out the treatments, the cell viability (Fig. S6A), caspase 3 activity, cell cycle, and migration ability of HEC-1-A cells were analyzed. Figure 5A was observed that LPPC-Q7 induced cleaved caspase 3 expressions of over 80% of cells for 6 h after removing the treatment, but Q7 alone did not induce cleaved caspase 3 in the cell and cause cell morphological changes (Fig. S6B). Moreover, LPPC-Q7 efficiently caused cells to arrest G0/G1 phase, but no obvious difference between Q7 and control groups (Fig. 5B). Furthermore, in migration assay (Fig. 5C), the assistance of LPPC could also rapidly provide the anti-migration effects of Q7 on HEC-1-A cells. The migration rate of LPPC-Q7 was significantly lower than Q7 alone.

### LPPC-Q7 with clinical drugs showed synergistic effects in HEC-1-A cells

**Figure 6:**
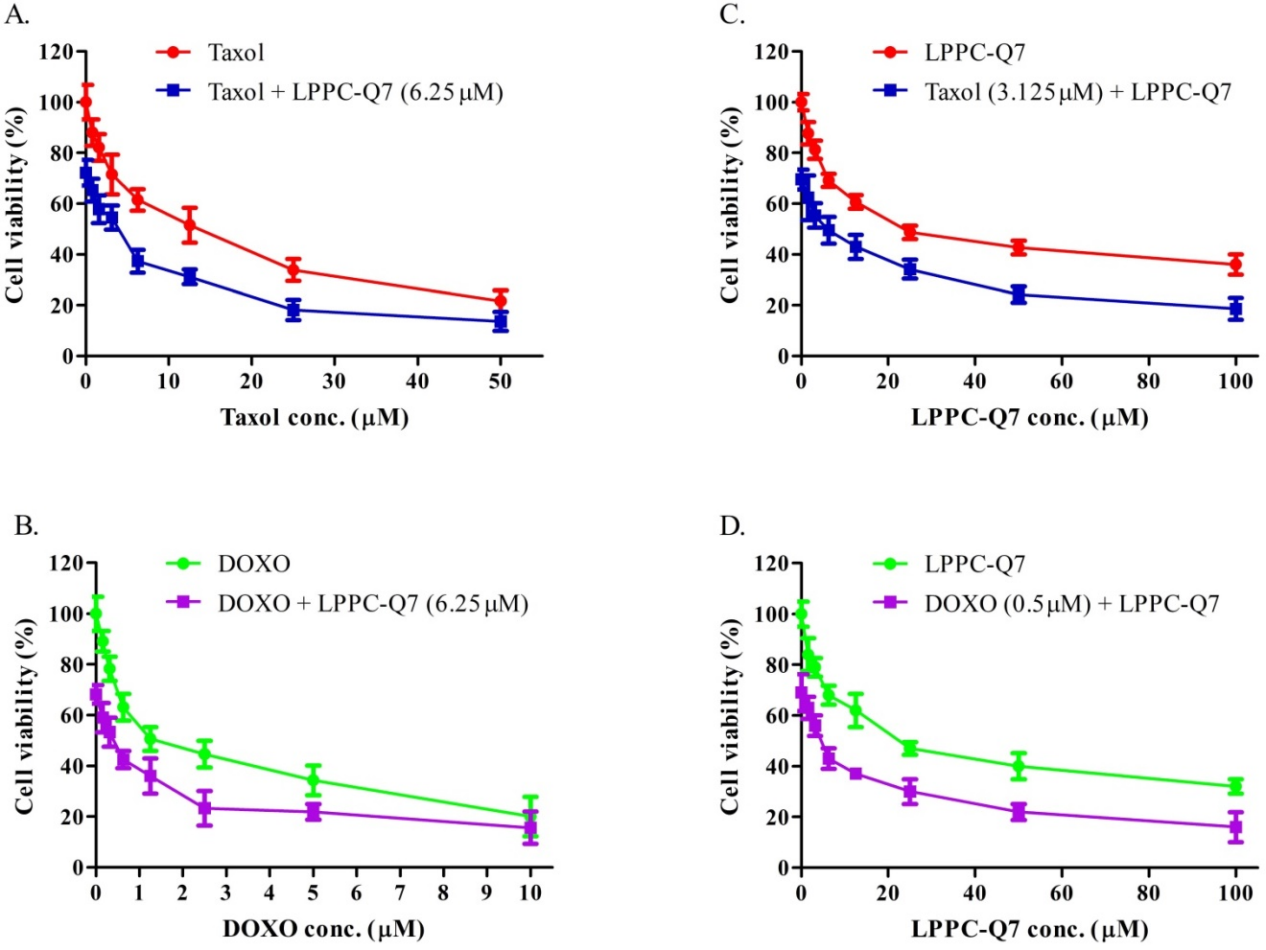
** LPPC-Q7 with paclitaxel (Taxol) or doxorubicin (DOXO) had a synergistic effect on HEC-1-A cells.** An MTT assay was determined that HEC-1-A cells were treated with LPPC-Q7 combined with paclitaxel (**A and C**) or doxorubicin (**B and D**).

According to the guidelines for the management of patients with endometrial carcinoma 41, doxorubicin and paclitaxel were both considered to be active drugs for chemotherapy. To determine the synergistic effect of LPPC-Q7 combined with paclitaxel or doxorubicin, the cell viability was analyzed. The results indicated that the inhibitory effects of drugs combined with different concentrations of LPPC-Q7 increased the cytotoxic effects in a dose-dependent manner (Fig. 6A and 6C). Similarly, when different concentrations of paclitaxel or doxorubicin were combined with LPPC-Q7, led to remarkable toxicity in the cells (Fig. 6B and 6D). The combination index (CI) of paclitaxel and LPPC-Q7 was lower than 1, and the CI value of the other combination was almost lower than 1 (Table S2). Therefore, the result demonstrated that LPPC-Q7 with paclitaxel synergistically inhibited the cell proliferation of HEC-1-A cells, and doxorubicin also did.

## Discussion

Endometrial cancer has a high incidence rate and is increasing yearly worldwide 1. Usually, the main treatment for endometrial cancer is surgery to remove the uterus and cervix. Despite advances in surgical resection, the prognosis of endometrial cancer is poor with a 5-year survival rate of < 40% 42. This operation area depends on the stages of cancer, and adjuvant therapy efficiently reduces tumor size to prevent from losing fertility 5-7. Anticancer peptides are natural kind of bioactive peptides which could be used as a novel type of anticancer drug that has several advantages over chemistry-based drugs, including high specificity, strong tumor penetration capacity, and low toxicity to normal cells 43, 44. Taken together, in this study, anticancer peptides Q7 was obtained from iDACP web tool and firstly demonstrated that could inhibit HEC-1-A cells proliferation, decrease migration ability, and cause cell death through the suppression of DHCR24 expression to mediate the AKT-modulated signal pathway. Moreover, utilized LPPC efficiently provided anticancer activities with low dosage Q7 and had a synergistic effect with clinical drugs on HEC-1-A cells (Fig. 5 and Fig. 6). Therefore, the results revealed that Q7 was another good treatment choice for endometrial cancer before surgery, especially complex with LPPC.

Malignant tumor cells have high cell proliferation rate and migration activity for over-growth and metastasis. Adjuvant therapy for pre-surgery patients could diminish operation areas, decrease metastatic tumor cells, reduce tumor relapse, and improve the overall survival rate 7. In this study, Q7 could suppress migration ability and cause cell death of HEC-1-A cells and provide efficient anti-tumor activities with the assistance of LPPC (Fig. 5 and Fig. 6). Furthermore, using LPPC could decrease over 6-fold dosage of Q7 to achieve fifty percentage growth inhibition of HEC-1-A cells (Fig. 4A). As previous studies, LPPC encapsulated several clinical drugs increased their cytotoxic activity of tumor drugs against tumor cells, and even drug-resistance cells also did 31. Therefore, Q7 had the potential for adjuvant therapy, and the anticancer activity of Q7 combined with LPPC could more efficiently suppress the malignancy of tumors, including metastasis and over-growth.

Currently, there are more than 60 US Food and Drug Administration (FDA)-approved peptide medicines on the market, and this is expected to grow significantly, with approximately 140 peptide drugs currently in clinical trials and more than 500 therapeutic peptides in preclinical development 45. However, several peptides have anticancer activity, and the clinical application of these peptide drugs is restricted due to poor pharmacokinetics and potential side effects 46. Besides, only limited *in vivo* studies showed that anticancer peptides alone could provide antitumor efficacy successfully 47, 48. Therefore, the challenges of anticancer peptide drugs are that proteolytic degradation of peptide drugs in serum limits the drug's half-life, fast elimination during the metabolic process, low membrane permeability, and reduces the bioavailable concentration 27. Several approaches have been utilized to overcome the drawbacks to increase the bioavailability of peptide drugs, such as liposome 29. A lipo-complex, LPPC, had been demonstrated rapidly delivered proteins into mice liver cells and retained their enzymatic activity *in vitro* and *in vivo* 32. Furthermore, we used LPPC that encapsulated doxorubicin and adsorbed Herceptin to specifically inhibit the growth of breast cancers *in vivo* 49. LPPC provided a better therapeutic efficacy in a xenografted model, and therefore we assumed LPPC-Q7 had the anticancer activity of Q7 in endometrial cancer *in vivo*.

DHCR24, the enzyme of cholesterol synthesis, is involved in several cellular functions, such as cell differentiation, anti-apoptosis, and anti-inflammation 50. Moreover, DHCR24 is dysregulated in many tumors, including endometrial cancer, prostate cancer, and ovarian cancer 9, 51, 52. The previous study indicated a significant correlation between DHCR24 overexpression and aggressive tumor behaviors in endometrial cancer, such as cell invasion and migration 9. Another study revealed that DHCR24 expression was associated with autophagy in Alzheimer's disease (AD) 53. We also evaluated autophagy marker LC3B expression of HEC-1-A cells with Q7 (Fig. S5). As the supplementary result showed that induction of LC3B was after 12 h treatment of Q7. DHCR24 could serve as a potential therapeutic target for the treatment of endometrial cancer.

Cholesterol-rich lipid rafts served as a framework for receptors and associated signaling factors, such as PI3K/AKT and RAS/RAF/MEK pathway 54, 55. In the study, we proved that Q7 decreased DHCR24 expression to interface AKT-kinase to induce caspase cascade to cause tumor cell apoptosis and mTOR-associated migration regulators. The composition of Q7 contained many amine groups, similar to PEI polymer, and the other study had shown that expression of AKT-kinase which was possibly one of the biomolecules affected by PEI 56. Other studies demonstrated polyamines, polycationic alkylamines, play a role in fundamental cellular processes including proliferation, apoptosis, and autophagy 57, 58. Moreover, RNA sequencing analysis helps us more comprehend the mechanism of Q7 (Fig. 2). Interestingly, the results showed that sQ7 had similar effects on HEC-1-A cells, such as cell proliferation (Fig. 1A) and migration (Fig. 1C). Similarly, the phosphorylation of AKT was also downregulated by the treatment of sQ7 in HEC-1-A cells (Fig. S3). These data proposed that sQ7 was able to interfere with AKT or other downstream regulators, like Q7. As the above discussion, the study also indicated that synthetic polypeptides containing repeated glutamine residues could act as intracellular enzymatic protein substrates 57.

The data were in line with previous literature showing the important role of DHCR24 in tumor growth and metastasis, which also indicated that DHCR24 might become an effective target in endometrial cancer therapy. To conclude, we demonstrated that Q7 caused cell arrest, induced cell apoptosis, and decreased the migration ability of HEC-1-A cells. And with the assistance of RNA-seq analysis, we efficiently proved the anti-tumor mechanisms of Q7 which could inhibit the tumorgenicity of HEC-1-A cells by suppressing DHCR24 expression to mediate the AKT-modulated signal pathway. Additionally, LPPC-Q7 had lower cytotoxicity in normal cells compared with HEC-1-A cells (Fig. 4). Moreover, LPPC-Q7 combined with doxorubicin or paclitaxel synergistically inhibited the growth of HEC-1-A cells, suggesting that clinical drugs combined with LPPC-Q7 could reduce the dose of drugs to moderate its adverse effects (Fig. 6). Therefore, LPPC-Q7 may be developed as a drug to reduce the occurrence of adverse events in endometrial cancer patients during chemotherapy, and furtherly possibly maintain their fertility without resecting the entire uterus. Finally, we believe that our findings contribute to obtaining a new anticancer peptide Q7 for endometrial cancer therapy.

## Supplementary Material

Supplementary figures and tables.

## Figures and Tables

**Table 1:** The IC50 values of anticancer peptides in HEC-1-A and HUVEC cell lines

ACP NO.	1	2	3	4	5	6	7	8	9	10
Cell line										
HEC-1-A	27.51±2.5	27.69±1.38	8.78±0.77	15.87±1.42	8.96±1.01	24.49±2.09	22.17±2.11	23.66±2.19	22.8±2.02	123.19±10.08
HUVEC	29.13±1.72	34.04±2.85	9.72±0.84	15.92±1.33	8.9±0.72	25.33±2.75	21.29±1.86	26.1±2.38	24.19±2.64	ND

The IC50 values revealed the concentration that results in a 50% decrease of cell viability. Values were the mean ± SD (μM) at 48 h. ND: no detection.

## Abbreviations

LPPC: Lipo-PEG-PEI-complex
NGS: next-generation sequencing
ACP: anticancer peptides
DHCR24: 24-Dehydrocholesterol Reductase
