# Supplementary material for: Anticancer peptide Q7 suppresses the growth and migration of human endometrial cancer by inhibiting DHCR24 expression and modulating the AKT-mediated pathway
Source: Int J Med Sci. 2022 Nov 7;19(14):2008–21. doi: 10.7150/ijms.78349 (PMC9724248; doi:10.7150/ijms.78349)
Supplement: Supplementary file 1 — Supplementary figures and tables. [file ijmsv19p2008s1.pdf]

Supplementary results

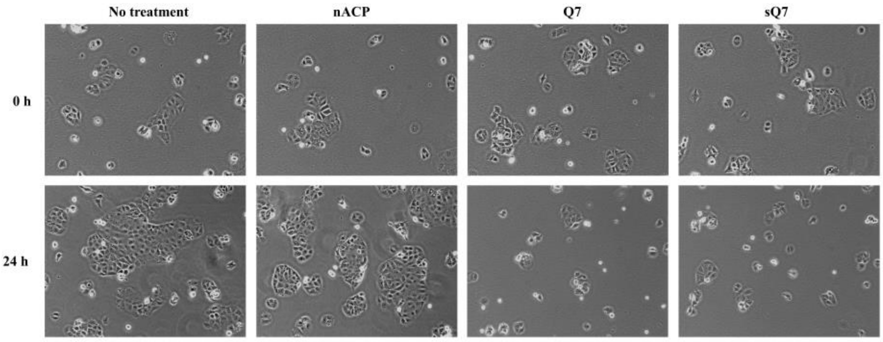

Figure S1. Anticancer peptide (Q7) inhibited HEC-1-A cells.

With or without treatments (peptides conc.: 100 μM) for 0 h (the upper) and 24 h (the lower), the representative photographs of cells were shown and observed under microscopy (100×).

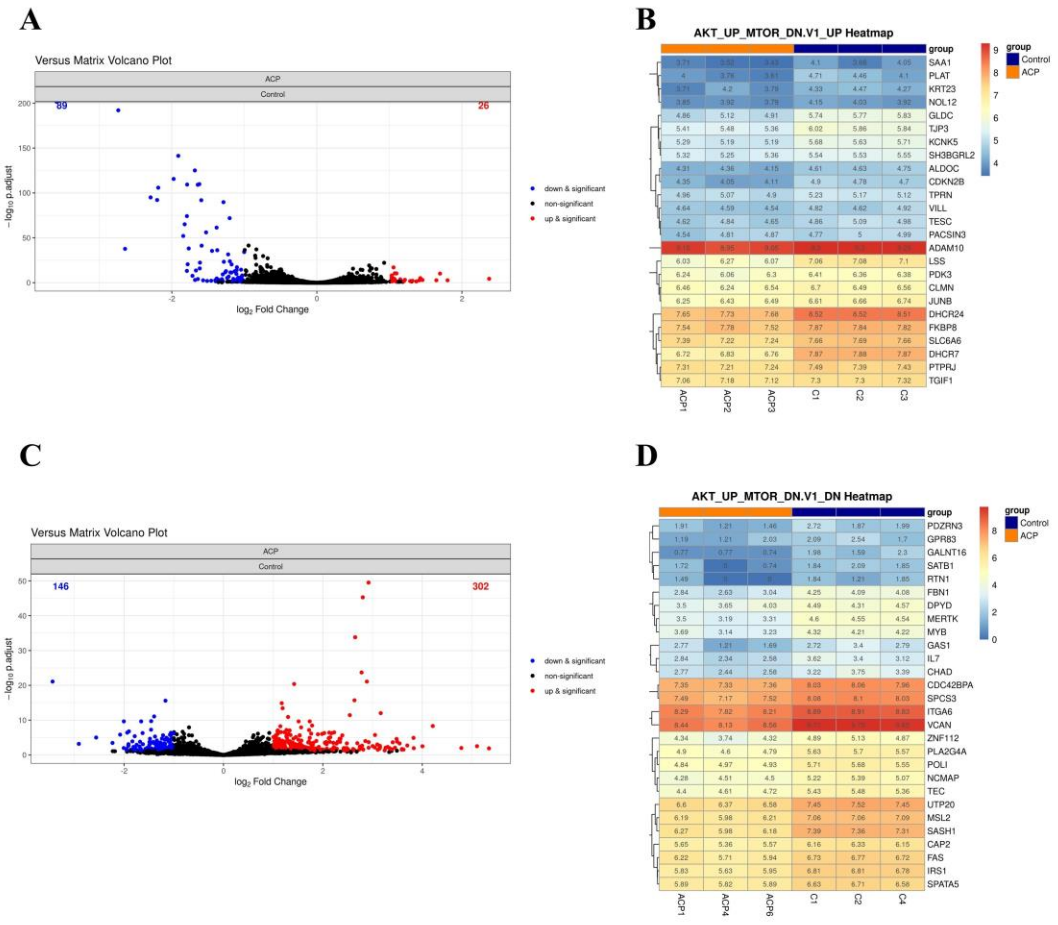

Figure S2. Volcano plots and heatmaps showed differentially expressed genes for different time of treatment.

A volcano plot was showed differentially expressed genes of HEC-1-A cells under Q7 treatment for 1 h (A) and 6 h (C), which significantly up and down-regulated genes were filtered (Log_2 Fold Change > ±1, p-value <.05 or p.adjust<.005) and highlighted in red and blue dots, respectively. Heatmaps exhibited significant differential genes involved in AKT/mTOR pathway for 1-h (B) and 6-h (D).

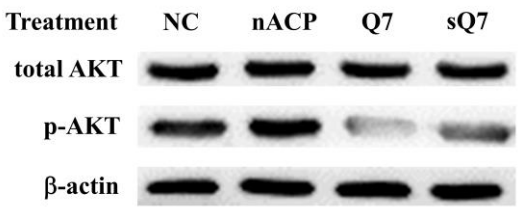

Figure S3. Q7 repressed the phosphorylation of AKT-kinase.

After 6 h incubation of Q7 (100 μM), sQ7 and nACP, the AKT protein expression of HEC-1-A cells was detected by using western blotting.

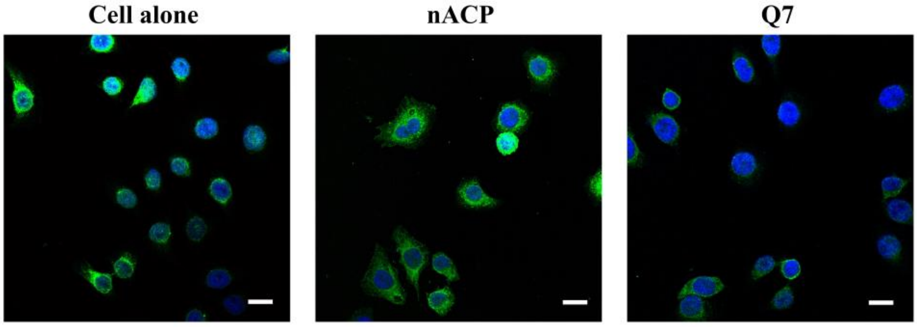

Figure S4. The expression of Caveolin 1 in HEC-1-A cells under Q7 treatment. After 2 h treatment (Q7, 100 μM), the cells were fixed with 4% paraformaldehyde for 15 min, permeabilized with 0.25% Triton X-100 for 10 min, and blocked with 5% BSA for 1 h at room temperature. The cells were labeled with Caveolin 1 rabbit polyclonal antibody (Thermo) at 1 μg/ml in 1% BSA and incubated for 3 h at room temperature and then labeled with goat anti-rabbit IgG (H+L) Superclonal™ secondary antibody, Alexa Fluor® 488 conjugate (Thermo) at a dilution of 1:2000 for 45 min at room temperature (green). Nuclei (blue) were stained with DAPI (Thermo). Scale bar: 20 μm.

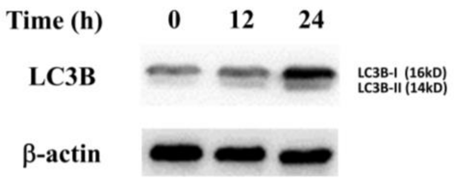

Figure S5. Q7 induced the expression of cell autophagy marker, LC3B, in HEC-1-A cells.

The LC3B protein expression was investigated HEC-1-A cells were treated with Q7 for different time points by using western blotting (0, 12, and 24 h).

A

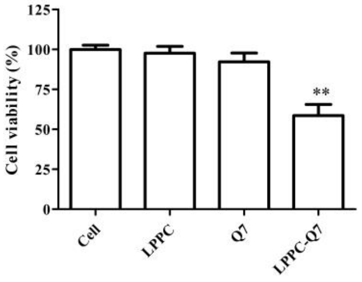

B

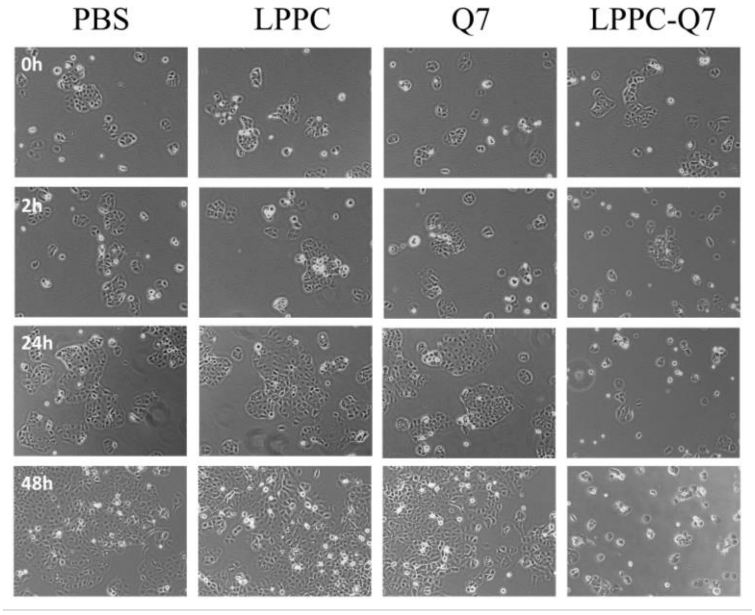

Figure S6. LPPC-Q7 efficiently causes cell death than Q7 alone treatment.

(A) Cell viability was determined that LPPC-Q7 provided the anticancer activities of Q7 in HEC-1-A cells. **: Indicated a significant difference between LPPC-Q7 and Q7 ($p<0.01$). (B) The representative photographs of HEC-1-A cells at different time points were showed and observed under microscopy (100×).

NO.	Sequence	anticancer mechanism	others
1	FKGGGPYGQGVTRGQDLSGKDF	Unknown	Unknown
2	AIPCGESCVWIPCISAAIGCSCKNKVCYR	Unknown	anti-HIV
3	MEFVAKLFKFFKDLLGKFLGNN	Unknown	strong lytic activity against human neutrophils
4	GSIPCGESCVWIPCISSVVGCACKNKVCYKN	probably disrupt membranes	plant defense mechanism
5	YKQCHKKGGHCFPKEKICIPPSSDFGKMDCRWRWKCCKKGSG	probably disrupt membranes	cell-penetrating
6	GVIPCGESCVFIPCISTLLGCSCKNKVCYRN	Unknown	plant defense mechanism; antibiotic activity; anti-HIV
7	KSCCPNTTGRNIYNTCRFGGGSREVCASLSGCKIISASTCPSYPDK	Unknown; probably toxic effect on the cell membrane	small plant proteins which are toxic to animal cells
8	GLLGVLGSVAKHVLPHVVPVIAEHL	disrupt membranes; TNF-α pathway	disrupt bacterial membranes
9	SVTPIVCGETCFGGTCNTPGCSCSWPICTK	probably disrupt membranes	plant defense mechanism
10	EQQQQQQPQNRRFRE	Unknown	Unknown

Table S1. The information of ten anticancer peptides from iDACP.

Taxol (μM)	LPPC-Q7 (μM)	CI value
50	6.25	0.44
25	6.25	0.35
12.5	6.25	0.47
6.25	6.25	0.39
3.125	6.25	0.64
1.5625	6.25	0.57
0.78125	6.25	0.75
3.125	100	0.35
3.125	50	0.33
3.125	25	0.41
3.125	12.5	0.46
3.125	6.25	0.48
3.125	3.125	0.53
3.125	1.5625	0.67

DOXO (μM)	LPPC-Q7 (μM)	CI value
10	6.25	0.69
5	6.25	0.6
2.5	6.25	0.35
1.25	6.25	0.44
0.625	6.25	0.4
0.3125	6.25	0.53
0.15625	6.25	0.6
0.5	100	0.29
0.5	50	0.29
0.5	25	0.33
0.5	12.5	0.34
0.5	6.25	0.36
0.5	3.125	0.58
0.5	1.5625	1.03

Table S2. CI value table list of different drug combinations.

The different treatments were defined as having an additive effect (CI = 1), synergism (CI < 1), or antagonism (CI > 1).
